# Whole-genome sequencing reveals an East Asian-specific rare variant of *INPP5J* associated with Alzheimer’s disease

**DOI:** 10.1038/s41398-026-04027-0

**Published:** 2026-04-08

**Authors:** Tetsuaki Kimura, Akiko Yamakawa, Risa Mitsumori, Shumpei Niida, Kouichi Ozaki, Daichi Shigemizu

**Affiliations:** 1 Medical Genome Center, Research Institute, National Center for Geriatrics and Gerontology, Obu, Aichi Japan; 2 Research Institute, National Center for Geriatrics and Gerontology, Obu, Aichi Japan; 3 Department of Aging Research, Nagoya University Graduate School of Medicine, Nagoya, Japan; 4 RIKEN Center for Integrative Medical Sciences, Yokohama, Japan; 5 Department of Cardiovascular Medicine, Hiroshima University Graduate School of Biomedical and Health Sciences, Hiroshima, Japan

**Keywords:** Medical genetics, Genomics

## Abstract

Late-onset Alzheimer’s disease (LOAD) is the most common form of dementia in the elderly, yet no curative treatments are available. Although genome-wide association studies (GWASs) have identified numerous genetic risk factors, these factors often differ among ethnic groups, and the mechanisms driving LOAD onset remain poorly understood. Most GWASs of LOAD have been conducted in European populations; the expansion of future studies to non-European populations should uncover novel genetic factors underlying LOAD pathogenesis. To identify novel LOAD-susceptible genes, we conducted whole-genome sequencing data analysis on 1928 Japanese individuals including 325 patients with LOAD and 1603 cognitively normal elderly controls. A GWAS for common variants identified a statistically significant association signal in rs429358, within the apolipoprotein E gene (*APOE*), which defines the *APOE*$\varepsilon$*4* haplotype. This association was successfully replicated in an independent Japanese replication cohort of 4768 samples, genotyped using the Asian Screening Array. For rare variants, a gene-based association study identified two rare variants, rs769490815 and rs1921732305, in *Inositol polyphosphate 5-phosphatase* (*INPP5J*) as potential candidates for LOAD association. Due to their extremely low allele frequencies, these variants were not included on the genotyping array and could not be evaluated in the replication cohort. However in vitro functional analyses revealed that the ethnicity-specific p.K687T mutation (rs1921732305) significantly reduced the phosphatase activity of INPP5J, suggesting a potential pathogenic role in LOAD.

## Introduction

Late-onset Alzheimer’s disease (LOAD) is the most common form of dementia in the older population and is an important global health challenge. Although current treatments may slow disease progression, no curative therapies have been established to date [1, 2]. A deeper understanding of the pathogenesis of LOAD is essential for the development of more effective treatments in the future.

LOAD is a complex disorder influenced by both genetic and environmental risk factors, with its heritability estimated to be approximately 60-80% [3, 4]. Genome-wide association studies (GWASs) have identified numerous genetic risk factors associated with LOAD [5], with the ε4 allele of the *apolipoprotein E* gene (*APOE*), located on chromosome 19, recognized as the strongest known genetic risk factor. However, this allele explains only approximately 25% of the estimated heritability [3], leaving a substantial portion of the genetic contribution unexplained. Therefore, further investigation is urgently needed to identify additional genetic contributors to the disease.

When the effect of the *APOE* ε4 allele on LOAD is accounted for, its impact varies across populations, being weaker in African populations and stronger in Japanese populations than in non-Hispanic Caucasians [6]. These findings highlight the population-specific genetic contributors to LOAD. Additionally, several population-specific LOAD-related genes have been identified. For instance, *GCH1*, *RHOBTB3*–*GLRX*, and *CHODL* have been reported in Han Chinese populations; *SUDS3*–*SRRM4* and *FAM47E*–*SCARB2* in Japanese populations; and *CHD2*, *CACNA1A*, and *LRIG1* in South Korean populations [7–11]. Given that most GWASs of LOAD have been conducted in European populations [5], the expansion of future studies to non-European populations should uncover novel genetic factors underlying LOAD pathogenesis.

In Japanese populations, several GWASs of common variants have identified population-specific genetic risk factors for LOAD [7, 8]. Moreover, a few studies utilizing whole-genome sequencing (WGS) have reported population-specific rare variants associated with LOAD [12] and dementia with Lewy bodies (DLB) [13]. However, these studies remain limited by their relatively small sample sizes (e.g., LOAD, n = 140; DLB, n = 61), highlighting the need for large-scale studies to validate and further explore these associations. Here, we conducted comprehensive association analyses of 325 LOAD patients, incorporating both common and rare variants, by using the largest Japanese WGS dataset for LOAD to date. Our analysis identified two rare coding variants of *inositol polyphosphate 5-phosphatase J* (*INPP5J*) as novel genetic risk-factor candidates for LOAD. Subsequent in vitro functional analyses revealed that the p.K687T mutation (i.e., rs1921732305) significantly reduced the enzymatic activity of INPP5J, suggesting its potential role in LOAD pathogenesis. We believe that these findings offer new insights into the mechanisms underlying LOAD and may contribute to future therapeutic strategies.

## Materials and methods

### Clinical samples

The National Center for Geriatrics and Gerontology (NCGG) Biobank holds DNA samples and the associated clinical data from tens of thousands of individuals, with WGS data available for thousands of participants. In this study, a total of 6761 samples were used. Of these, WGS data were available for 1993 samples, including 336 LOAD patients and 1657 cognitively normal (CN) older adults. The remaining 4768 DNA samples, comprising 1732 LOAD patients and 3036 CN subjects, were used for the subsequent replication study. Donors with LOAD were diagnosed by using the criteria of the National Institute on Aging Alzheimer’s Association workgroups [14, 15]. The CN subjects had subjective cognitive complaints but demonstrated normal cognition as determined by a comprehensive neuropsychological assessment, including a Mini-Mental State Examination score > 23. All subjects were diagnosed by using medical history, physical and neurological examinations, neuropsychological tests, and brain imaging with either MRI (magnetic resonance imaging) or CT (computed tomography).

### WGS data analysis

All WGS data were downloaded from the NCGG Biobank database and the NCGG Integrated Database for Dementia Research (iDDR). A WGS library was prepared by using a TruSeq DNA PCR-Free Library Preparation Kit (Illumina Inc., San Diego, CA, USA) in accordance with the manufacturer’s protocol. WGS was performed at Takara Bio Inc. (Shiga, Japan), with DNA sequencing conducted by using NovaSeq 6000 platforms (Illumina) with paired-end reads of 151 bp, in accordance with the manufacturer’s protocol.

Read sequences were mapped to the human reference genome (GRCh37) by using BWA-MEM (version 0.7.15) [16]. Duplicate polymerase chain reaction (PCR) reads were removed with Picard (version 2.21.4) [17]. Variant calling was conducted by using a Genome Analysis Toolkit (GATK: version 4.1.0.0) [18]. Individual variant calling was performed with GATK HaplotypeCaller, and multi-sample individual variants were called via joint calling with in-house other WGS data by using GATK GenotypeGVCFs [18]. Variant quality-score recalibration was performed in accordance with GATK Best Practice recommendations. We filtered out single nucleotide variants (SNVs) that satisfied the following criteria: Depth < 10, GenotypeQuality < 20, Quality by Depth < 2, QUAL < 30, StrandOddsRatio > 4, FisherStrand > 60, RMSMappingQuality (MQ) < 40, MappingQualityRankSumTest < −12.5, ReadPosRankSumTest < −8, and Excess Heterozygosity > 20. Additionally, short insertions and deletions (indels) were filtered out by using the following criteria: Depth < 10, GenotypeQuality < 20, Quality by Depth < 2, QUAL < 30, StrandOddsRatio > 10, FisherStrand > 200, ReadPosRankSumTest < −20, and Excess Heterozygosity > 20.

Quality control (QC) of these variants was performed by using PLINK software [19]. We first applied QC filters to the subjects for sex inconsistencies (--check-sex), PI_HAT > 0.25, where PI_HAT is a statistic for the proportion of identity by descent (--genome), genotype missingness (--mind 0.05), inbreeding coefficient (--het 0.1), and exclusion of outliers from the clusters of East Asian populations in a principal component analysis that was conducted by using the 1000 Genomes Phase 3 data [20]. We next applied QC filters to the variants for genotyping efficiency or call rate (--geno 0.05), minor count (--mac 2), and Hardy–Weinberg equilibrium (--hwe 1 × 10^−5^).

### Variant annotation

Functional annotations of the variants were conducted by using ANNOVAR (version 20191024) [21]. We used protein-coding variants such as frameshift indels, nonframeshift indels, stop-gain/-loss variants, nonsynonymous and synonymous SNVs, and splicing variants. Variant frequency data were obtained from the ToMMo 54KJPN database [22]. Because the sample size in this study (n = 1928) is relatively modest for reliable estimation of rare variant allele frequencies, we used the database, which comprises a much larger Japanese whole-genome sequencing reference panel (n = 54000). This approach provides more stable and accurate population-specific allele frequencies, particularly for rare variants. On the basis of the minor allele frequencies (MAFs), variants were classified into two categories: common variants (MAF $\ge \,$0.01) and rare variants (MAF < 0.01).

### Common and rare variant analyses

A GWAS of common variants was conducted by using logistic regression, adjusting for sex and age, with PLINK software (--logistic). Candidate pathogenic variants that met the genome-wide significance threshold (*P* < 5.0 × ${10}^{-8}$) were genotyped in an independent replication cohort. Association analyses were performed not only in the replication cohort, but also in the combined dataset of the WGS and replication cohorts, by using PLINK software (--logistic) with adjustments for sex and age. Linkage disequilibrium (LD) information and population recombination rates for the association signals were obtained using the LocusZoom (http://locuszoom.org/). The genomic control inflation factor was calculated by using PLINK.

Rare variant analysis focused on the coding of rare variants with a Combined Annotation Dependent Depletion Phred-scaled score (CADD score) ≥ 20, corresponding to the top 1% of predicted deleterious variants in the human genome. Gene-based association tests—specifically the optimal sequence kernel association tests (SKAT-O), exome-based rare variant association approach designed to evaluate whether rare variants within specific genes contribute to the development of phenotypic variation [23]—were conducted by using R programming language (R Development Core Team, http://www.r-project.org/). SKAT-O analyses were performed for genes harboring at least two rare variants with CADD ≥ 20. Genes with a Bonferroni-corrected *P* < 0.05 were considered to be significantly enriched. Samples carrying the minor allele were validated using Sanger sequencing.

### Prediction of inositol polyphosphate-5-phosphatase J (INPP5) protein structures

The domain structures of INPP5D (NM_001017915.3), INPP5J (NM_001284285.2), and INPP5K (NM_016532.4), were analyzed by using InterProScan [24]. The 3D structure of INPP5J was predicted by using AlphaFold2 with MMseqs2 (ColabFold version 1.5.5) [25], and the resulting structure was visualized by using PyMOL (version 2.5.0) [26].

### Cloning of *INPP5J*

The cDNA clone of *INPP5J* (HG13987-ANR), purchased from Sino Biological (Beijing, China), contained a substitution at amino acid position 791 of the full-length *INPP5J* sequence (NM_001284285), where tryptophan was replaced with arginine. This substitution was corrected by using a KOD-Plus-Mutagenesis Kit (Toyobo Co., Ltd., Osaka, Japan) in accordance with the manufacturer’s protocol. Additionally, as this clone lacked the 5ʹ sequence (1138 bp), making it shorter than the coding sequence of NM_001284285, the missing 5ʹ sequence was synthesized and integrated into the corrected clone by PCR. The full-length *INPP5J* coding sequence was then cloned into a pT7CFE1-NHis-GST-CHA vector (Thermo Fisher Scientific Inc., Waltham, MA, USA) by using In-Fusion Snap Assembly Master Mix (Takara Bio Inc.). Both the p.R15W mutation and the p.K687T mutation were introduced by using a KOD-Plus-Mutagenesis Kit. The sequences of all clones were confirmed by using an ABI sequencer (Thermo Fisher Scientific Inc.). Information on the primers used is listed **(**Table S1**)**.

### Phosphatase activity assay of INPP5J proteins

Wild-type (WT), p.R15W, and p.K687T INPP5J proteins were synthesized in vitro by using a 1-Step Human High-Yield IVT Kit (Thermo Fisher Scientific Inc.), purified with glutathione agarose, and eluted by HRB3C protease cleavage (Thermo Fisher Scientific Inc.), in accordance with the manufacturer’s protocol. Protein concentrations were measured with a TaKaRa Bradford Protein Assay Kit (Takara Bio Inc.), and protein synthesis was confirmed by sodium dodecyl-sulfate polyacrylamide gel electrophoresis (SDS-PAGE) using Mini-PROTEAN TGX Gels 4–20% (Bio-Rad Laboratories, Inc., Redmond, WA, USA). The molecular-weight markers used were Precision Plus Protein Dual Xtra Standards (Bio-Rad Laboratories, Inc.).

Phosphatase activity assays were performed by incubating 50 nM of the INPP5J WT or the p.R15W or p.K687T mutant with 40 μg/mL phosphatidylinositol 3,4,5-trisphosphate (PI(3,4,5)P_3_) diC8 at 37 °C for 1 h, in accordance with the method described by Dong et al. [27]. Phosphate release was measured with a PhosphoWorks Fluorimetric Phosphate Assay Kit *Red Fluorescence* (AAT Bioquest Inc., Pleasanton, CA, USA) in accordance with the manufacturer’s protocol. A total of four independent experiments were conducted. The difference in phosphatase activity levels between WT and the mutants was assessed by using Welch’s *t*-test, with a *P* value of 0.05 or less considered statistically significant.

## Results

### WGS of Japanese individuals

We performed a WGS analysis of a total of 1993 Japanese individuals, consisting of 336 LOAD patients and 1657 CN subjects, by using the Illumina NovaSeq 6000 platforms. On average, 364 million read pairs were obtained from the analysis, of which 98.3% were mapped to the human reference genome (GRCh37), and 8.1% were removed as PCR duplicates. A total of 47,999,850 genetic variants were detected (i.e., SNVs and indels). Of them, 9,460,685 genetic variants from 1928 samples (325 LOADs and 1603 CNs) passed stringent QC criteria for both genotypes and samples (see Methods, Table S2 and Fig. S1).

The genetic variants were classified into 7,369,211 common (77.89%) and 2,091,474 rare (22.11%) variants on the basis of the MAFs obtained from the ToMMo 54KJPN database. The common variants consisted of 7,320,726 non-coding and 48,485 coding variants. Among the rare variants there were 2,070,606 non-coding and 20,868 coding variants. To identify novel LOAD-associated variants in Japanese subjects, we conducted a GWAS for common variants and a gene-based association study for rare variants.

### Association study for common variants

**Fig. 1:**
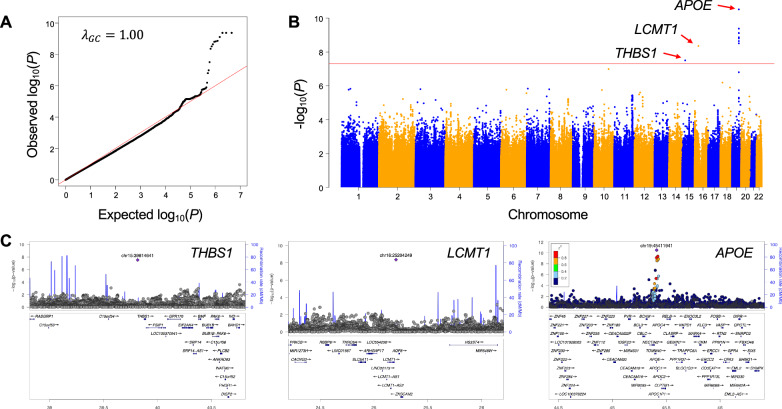
Results of a genome-wide association study (GWAS) using common variants. **A** Quantile-quantile plot of late-onset Alzheimer’s disease (LOAD) GWAS. The red line represents the expected values under the null hypothesis for no association. **B** Manhattan plot of common variants for genome-wide association with LOAD. The threshold for genome-wide significance (*P* < 5 × 10^−8^) is indicated by the red line. Three genomic regions (*THBS1*, *LCMT1*, and *APOE* regions) showed significant LOAD associations. **C** Regional association plots for the variants in the *THBS1*, *LCMT1*, and *APOE* loci, presented from left to right. Purple diamonds indicate the single nucleotide variant at each locus that exhibited the strongest association.

A GWAS for common SNVs was conducted by using logistic regression, adjusted for age and sex. The genomic control inflation factor was 1.00 (Fig. 1A). Three genomic regions reached the GWAS significance threshold of *P* < 5.0 × ${10}^{-8}$ (Fig. 1B, C). Among them, rs429358, which is an ε4 polymorphism in the protein encoded by *APOE*, exhibited the strongest significant association signal, with a *P* value of 3.06 × 10^−11^ and an odds ratio of 2.32 (Table 1).

**Table 1:** Results of genome-wide association study of common variants.

Variant (nearest gene)	Chromosome	Allele	Stage (n)	Genotype (A1/A1A2/A2)		A1 allele frequency		OR	95% CI	*P* value
	position	A1/A2		AD	CN	AD	CN			
rs429358 (*APOE*)	chr19 45411941	C/T	**GWAS (1926)**	**24/90/211**	**11/285/1305**	**0.21**	**0.096**	**2.32**	**1.81–2.98**	**3.06** × **10**^**–11**^
			**Replication (4768)**	**117/670/945**	**27/514/2495**	**0.26**	**0.094**	**5.36**	**4.64–6.20**	**5.25** × **10**^**–114**^
			**Combined (6694)**	**141/760/1156**	**38/799/3800**	**0.25**	**0.094**	**3.97**	**3.55–4.44**	**5.70** × **10**^**–128**^
rs966460365 (*LCMT1*)	chr16 25204249	A/T	**GWAS (1847)**	**0/28/290**	**0/16/1513**	**0.044**	**0.005**	**8.52**	**4.17–17.42**	**4.32** × **10**^**–9**^
			Replication (4768)	2/79/1651	1/138/2897	0.024	0.023	1.00	0.73–1.36	0.98
			Combined (6615)	2/107/1941	1/154/4410	0.027	0.017	1.68	1.29–2.19	1.23 × 10^–4^
rs4923834 (*THBS1*)	chr15 39814641	C/T	**GWAS (1871)**	**0/15/287**	**8/329/1232**	**0.025**	**0.11**	**0.22**	**0.12–0.38**	**3.09** × **10**^**–8**^
			Replication (4768)	16/346/1370	42/557/2437	0.11	0.11	1.05	0.90–1.22	0.55
			Combined (6639)	16/361/1657	50/886/3669	0.097	0.11	0.86	0.75–0.98	0.022

Bold font indicates statistical significance. *P* values are adjusted for age and sex by logistic regression analysis.

*GWAS* genome-wide association study, *AD* Alzheimer’s disease, *CN* cognitively normal older adults, *OR* odds ratio, *CI* confidence interval.

To validate these association signals, we analyzed an independent Japanese replication cohort of 4768 samples (1732 LOAD patients and 3036 CN subjects), genotyped using the Asian Screening Array (ASA). In this cohort, rs429358 remained significantly associated with LOAD, whereas the remaining two, rs966460365 and rs4923834, did not show significant associations (Table 1). Finally, a meta-analysis combining the GWAS discovery and replication cohorts confirmed that only rs429358 was significantly associated with LOAD (Table 1).

### Association study for rare variants

We functionally annotated rare coding variants and selected potentially deleterious mutations, including frameshift indels, splicing variants, stop-gain/stop-loss variants, and nonsynonymous SNVs, with CADD scores of at least 20. By using these deleterious variants, we conducted a genome-wide gene-based SKAT-O test. Of the 1,066 genes, one gene, *INPP5J*, reached Bonferroni-corrected significance (*P* < 0.05; corrected *P* = 0.032) (Table S3). Two rare variants in *INPP5J* were involved in these associations. The two *INPP5J* variants showed significant associations with LOAD in a logistic regression model adjusted for age and sex (Table 2). Notably, no patients were observed to be simultaneously heterozygous for both *INPP5J* variants. Because the allele frequencies of these rare variants were extremely low, they were not included the ASA genotyping platform and therefore could not be evaluated in the replication cohort.

**Table 2:** Association analysis of rare variants.

Gene	Variant	Allele	Allele (A1/A1A2/A2)		A1 frequency		OR	95% CI	*P* value
		A1/A2	AD	CN	AD	CN			
***INPP5J***	**rs769490815**	**T/C**	**0/4/262**	**0/1/1592**	**0.0075**	**0.00031**	**70.17**	**1.76–2803**	**0.024**
***INPP5J***	**rs1921732305**	**C/A**	**0/4/321**	**0/3/1594**	**0.0062**	**0.00094**	**5.78**	**1.22–27.42**	**0.027**

Bold font indicates statistical significance. *P* values are adjusted for age, sex, and *APOE* ε4 status by logistic regression analysis.

*AD* Alzheimer’s disease, *CN* cognitively normal older adults, OR odds ratio, *CI* confidence interval.

To investigate population-specific differences in allele frequencies, we examined the two *INPP5J* variants by using the gnomAD (https://gnomad.broadinstitute.org/) and ToMMo 54KJPN [28] databases (Table 3). Although rs769490815 was detected in other populations, it was observed more frequently in the East Asian population. In contrast, rs1921732305 was not detected in any other populations. These association signals with LOAD appeared to be specific to East Asians.

**Table 3:** Minor allele frequencies of two *INPP5J* variants in different populations.

rsID	NCGG biobank (Japanese)			ToMMo 54KJPN	gnomAD v.4.1.0			
	AD	CN	All		East Asian	South Asian	African/African American	European (non-Finnish)
rs769490815	0.0075	0.00031	0.0013	0.0005	2.30 × 10^–4^	9.34 × 10^–5^	2.94 × 10^–5^	8.94 × 10^–7^
rs1921732305	0.0062	0.00094	0.0018	0.0011	3.60 × 10^–4^	0.00	0.00	0.00

*AD* Alzheimer’s disease, *CN* cognitively normal older adults.

### Functional annotations of the *INPP5J* variants

**Fig. 2:**
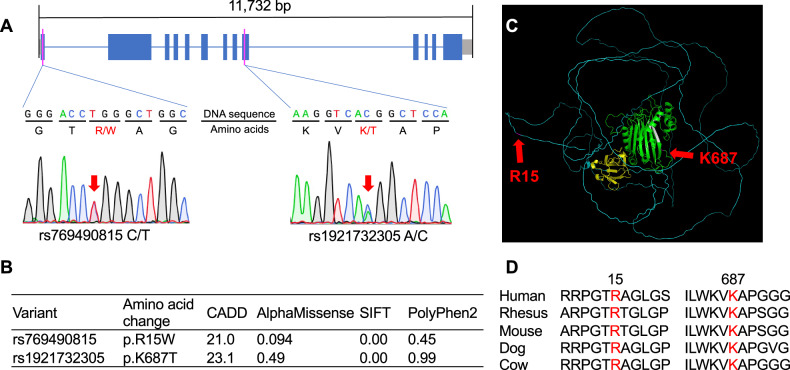
*INPP5J* variants identified in rare variant association studies. **A** Schematic representation of *INPP5J*, highlighting the locations of two variants. The mRNA was transcribed from left to right. Blue boxes represent coding regions, gray boxes represent untranslated regions (UTRs), and solid lines represent introns. Pink vertical lines indicate the positions of the variants rs769490815 and rs1921732305. Sanger sequencing results of samples with heterozygous variants are shown, with red arrows indicating the validated variant positions. Amino acids with substitutions are indicated in red. **B** Functional predictions of two variants. **C** Predicted three-dimensional structure of the INPP5J protein. The green, white, and yellow regions represent the phosphatase domain, its conserved active center, and the SKICH domain, respectively. Red arrows indicate the positions of mutated amino acids. **D** Both variant sites are highly conserved among mammals, with red letters indicating the amino acids replaced by the mutations. Numbers indicate the positions of the amino acids.

The two *INPP5J* variants, rs769490815 and rs1921732305, were nonsynonymous SNVs, resulting in p.R15W and p.K687T, respectively (Fig. 2A, B). R15 was located outside the domain structure, whereas K687 resided within the phosphatase domain (Fig. 2C). The AlphaMissense scores suggested that these variants were unlikely to affect the structural integrity of the protein (Fig. 2B). However, both variants have CADD scores $\ge$ 20, placing them among the top 1% of deleterious variants in the human genome. Additionally, their SIFT (sorting intolerant from tolerant) scores classified them as deleterious, and their PolyPhen2 (polymorphism phenotyping v2) scores categorized them as possibly or probably damaging (Fig. 2B). Furthermore, both variant sites are highly conserved among mammals (Fig. 2D), suggesting that they have potential functional significance.

### Functional analysis of the *INPP5J* variants

**Fig. 3:**
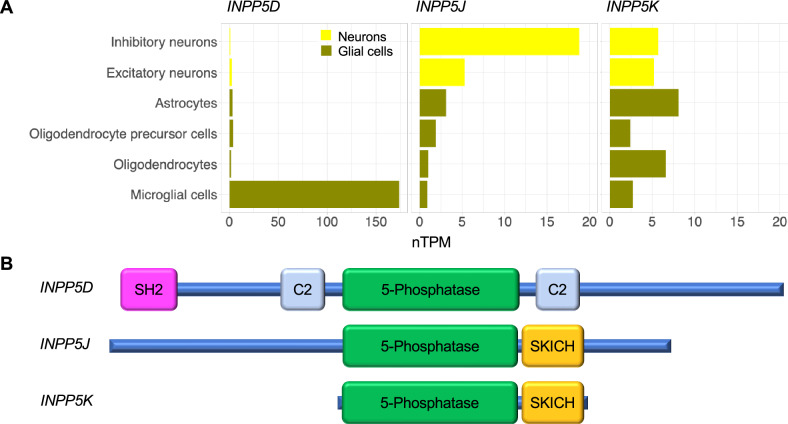
Expression patterns in the brain and domain structures of *INPP5D*, *INPP5J*, and *INPP5K.* **A** Expression patterns of *INPP5D*, *INPP5J*, and *INPP5K* in the brain. Numbers on the x-axis represent normalized transcripts per million (nTPM) obtained from the Human Protein Atlas database. **B** Domain structures of INPP5D, INPP5J, and INPP5K. INPP5J and INPP5K contain the SKICH domain (yellow box), which mediates plasma membrane localization. INPP5D has a domain structure different from those of the other two proteins. Blue bars indicate regions without a defined domain structure. Boxes indicate domains. SH2, Src homology 2 domain.

INPP5J is a member of the human PI 5-phosphatase family [29]. It has been reported as a phosphatase that removes the 5ʹ-phosphate from PI(3,4,5)P_3_ [30]. Another member of this family, INPP5D, is reportedly associated with LOAD by functioning in microglia within the brain [31]. However, whereas *INPP5D* is expressed in the microglial cells of the brain, *INPP5J* is expressed primarily in neurons, according to the Human Protein Atlas database [32] (Fig. 3A). This suggests that INPP5J and INPP5D have distinct functions in the brain.

Consideration of the structure of functional domains within the INPP5 family reveals that INPP5J has a domain structure similar to that of INPP5K (Fig. 3B). Both encoding genes are also expressed in brain neurons (Fig. 3A). These similarities suggest that INPP5J and INPP5K may have related roles in neuronal signaling pathways. Homozygous mutations in the phosphatase domain of INPP5K are associated with reduced enzyme activity, leading to congenital muscular dystrophy with cataracts and mild cognitive impairment [33–36]. Therefore, our findings of heterozygous *INPP5J* mutations, if associated with reduced enzyme activity, may also contribute to cognitive decline, similar to the case with *INPP5K*.

**Fig. 4:**
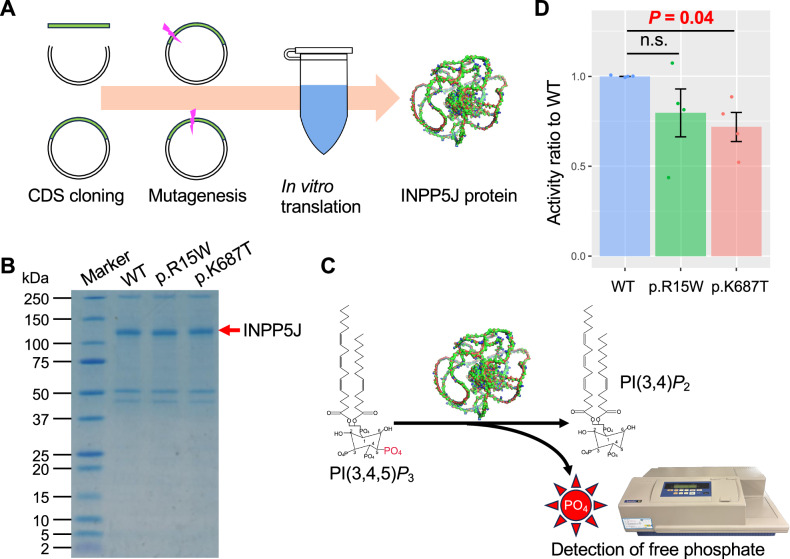
Phosphatase activities of the INPP5J mutants. **A** Scheme of in vitro translation of INPP5J. **B** Photograph of SDS-PAGE of purified INPP5J proteins. Coomassie brilliant blue staining detected the purified INPP5J with Linker and HA-tag (111 kDa) (red arrow). Molecular-weight markers were Precision Plus Protein Dual Xtra Standards (Bio-Rad Laboratories, Inc., Redmond, WA., USA). Numbers to the left of the marker lane indicate the sizes of the bands in kilodaltons. **C** Schematic diagram of free phosphate concentration measurement. **D** Phosphatase activity assay using purified INPP5J proteins. The phosphatase activities of the INPP5J mutants (p.R15W and p.K687T) were compared with that of the wild type (WT) by using Welch’s *t-*test. n.s. indicates no significant difference.

To test this hypothesis, we examined the phosphatase activity of the *INPP5J* mutants (p.R15W and p.K687T) and the WT protein. These three proteins were synthesized via in vitro translation (Fig. 4A, B), and free phosphate release was quantified by incubating each protein with the substrate PI(3,4,5)P_3_ (Fig. 4C). The p.K687T mutant exhibited significantly lower activity than the WT (Welch’s *t-*test *P* = 0.04, Fig. 4D), whereas the p.R15W mutant showed no significant difference (Welch’s *t*-test *P* = 0.22, Fig. 4D). These findings suggest that the p.K687T mutation (rs1921732305) may impair INPP5J phosphatase function, potentially contributing to LOAD development.

## Discussion

We investigated novel genetic risk factors for LOAD by using the largest Japanese WGS dataset to date. Our study identified two genes associated with LOAD: the well-established *APOE* and a novel candidate, *INPP5J*, identified through association studies of common and rare variants, respectively. The association signal for *INPP5J* corresponds to an East Asian-specific missense variant. Subsequent in vitro functional analyses revealed that this variant reduces the phosphatase activity of INPP5J, suggesting that it has a potential pathogenic role in the development of LOAD.

*INPP5J* is one of the 10 human PI 5-phosphatase family genes [29] and encodes a PI 5-phosphatase that utilizes PI(3,4,5)P_3_ as a substrate [37]. Another member of this family, *INPP5D*, plays crucial roles in the progression of LOAD through processes such as synaptic engulfment [38], cytokine release, and amyloid-beta phagocytosis/endocytosis [39]. Reduced activity of *INPP5D* has been shown to alter the microglial response to amyloid beta plaques [40–42], establishing it as a key LOAD risk gene with a crucial role in microglial function. Whereas *INPP5D* is highly expressed in microglia, *INPP5J* is expressed predominantly in neurons [32]. Inhibition of *Inpp5j* function causes abnormal neurite outgrowth in rats [30, 43]. INPP5J is localized to membrane ruffles [44], where it is believed to be involved in micropinocytosis (a form of endocytosis) through its regulation of PI(3,4,5)P_3_ metabolism. Given that neuronal endocytosis is implicated in LOAD pathogenesis [45, 46], the *INPP5J* mutation identified here may impair neuronal function in the brain, thereby contributing to the development of LOAD.

Further evidence supporting the involvement of *INPP5J* in the onset of LOAD comes from the identification of a loss-of-function mutation in *INPP5K* that causes congenital muscular dystrophy with mild cognitive impairment. Among the PI 5-phosphatase family genes, *INPP5J* and *INPP5K* share the highest degree of structural similarity [29]. Additionally, mutations in *OCRL—*another member of the PI 5-phosphatase family—cause Lowe syndrome, which is characterized by congenital cataracts, intellectual disability, and proximal renal tubular dysfunction [47]. Furthermore, overexpression of *SYNJ1—*which encodes another PI 5-phosphatase—in the brain has been linked to LOAD pathology and cognitive decline [48–50]. These findings suggest that the reduced phosphatase activity of INPP5J may contribute to cognitive impairment and play a pathogenic role in LOAD.

The p.K687T mutation significantly decreased phosphatase activity, while another mutation, p.R15W, showed a trend toward decreased phosphatase activity that did not reach statistical significance. These findings suggest that decreased INPP5J phosphatase activity may contribute to AD pathogenesis. Loss of INPP5J function is predicted to increase levels of PI(3,4,5)P3 and decrease levels of PI(3,4)P2. However, elevated PI(3,4,5)P3 levels is likely to be rapidly depleted by PTEN. Consequently, the levels of PI(3,4,5)P3 and PI(3,4)P2—key scaffolds for AKT phosphorylation—may be reduced, leading to suppressed AKT activation and subsequent activation of glycogen synthase kinase 3 β (GSK3β). Activation of GSK3β promotes to abnormal tau phosphorylation in neurons, facilitating neurofibrillary tangle formation and contributing to AD development [51]. Future studies are necessary to directly evaluate the effects of each *INPP5J* mutation on AKT and GSK3β activity, as well as tau phosphorylation, using iPS cell-derived neuronal models.

In this study, we were able to assess only the effects of the two mutations on INPP5J phosphatase activity. The p.R15W variant, which did not show a clear reduction in enzymatic activity, is located outside the phosphatase domain and may instead modulate INPP5J function through protein-protein interactions. In fact, INPP5J has been reported to forms a complex with DPYSL2/CRMP2, which exerts opposite effects on neurite elongation [43]. Further studies are needed to determine whether the p.R15W mutation alters enzymatic activity or downstream signaling in neuronal contexts.

We conducted WGS of a large cohort of Japanese subjects and performed LOAD association analyses incorporating both common and rare variants. Our analysis identified two ethnicity-specific rare variants of *INPP5J* as potential candidates for LOAD association. Functional analyses revealed that the p.K687T variant of *INPP5J* leads to reduced phosphatase activity, which may have implications for the pathogenesis of LOAD. As *INPP5K—*a paralog of *INPP5J—*has been implicated in brain abnormalities through reduced phosphatase activity, the p.K687T variant is likely to contribute to the onset of LOAD. We believe that our findings offer novel insights into the molecular mechanisms underlying LOAD and could pave the way for the development of targeted therapeutic strategies.

## Supplementary information

supplemental_materials

## Data Availability

Data are available from the corresponding author upon request.

## Code availability

We used the open source program languages R (version 3.4.3) and Ruby (version 2.4.0) to analyze the data and create the plots. Code is available upon request from the corresponding authors.
