# Supplementary material for: Whole-genome sequencing reveals an East Asian-specific rare variant of INPP5J associated with Alzheimer’s disease
Source: Transl Psychiatry. 2026 Apr 8;16:267. doi: 10.1038/s41398-026-04027-0 (PMC13183990; doi:10.1038/s41398-026-04027-0)
Supplement: Supplementary file 1 — supplemental_materials [file 41398_2026_4027_MOESM1_ESM.pdf]

Table S1. Primers used in this study

Primer	Sequence	Experiment
rs769490815-L	GCCTCTTCCAGGCTGGG	Sanger sequencing
rs769490815-R	GTGGAACCAGGGATCAGGA	Sanger sequencing
rs1921732305-L	CAAGGTGGAGGGTTACATCTTG	Sanger sequencing
rs1921732305-R	GAGGATGAGGCCAGAACTCA	Sanger sequencing
rs143017607-F	AGCTGAGAGCACTGAGGACCT	Sanger sequencing
rs143017607-R	GAGCTGACAGCTGAGGGAGTC	Sanger sequencing
pT7CFE1-up	CATATGGTTATCGGGCCCCTGGAA	In-Fusion cloning
pT7CFE1-dw	GGATCCGAATTCGATATCTTAATTAAGCTGCAG	In-Fusion cloning
Full-insert-L	CCCGATAACCATATGGAGGGCCAGAGCAGCAGG	In-Fusion cloning
Full-insert-R	ATCGAATTCGGATCCGGGCCCCAGGCCCCCTT	In-Fusion cloning
INPP5J-upL	ATGGAGGGCCAGAGCAGCAGG	INPP5J 5'-CDS Cloning
INPP5J-dwR	GCCCTGTACTCTGTGTGCCAAGCCT	INPP5J 5'-CDS Cloning
HG13987-ANR-L	GCCCTCCCAAGGCTTGGCAC	INPP5J CDS Cloning
HG13987-ANR-R	GGGCCCCAGGCCCCCTT	INPP5J CDS Cloning
R15W-up	AGGTCCCTGGCCTCCTGCTGC	INPP5J CDS Mutagenesis
R15W-dw	GGGCTGGCCTGGGTTCCC	INPP5J CDS Mutagenesis
K687T-up	GTGACCTTCCATAGGATACGGTCTGTCC	INPP5J CDS Mutagenesis
K687T-dw	GGCTCCAGGTGGGGGTCCC	INPP5J CDS Mutagenesis
W781R-up	AGACATAAGCCACATAGTCCTTGCAATGG	INPP5J CDS Mutagenesis
W781R-dw	GGGCCAAACATGAAGATGTGGATG	INPP5J CDS Mutagenesis

Abbreviation: CDS, coding DNA sequence

Table S2. Results of sample QC

QC criteria	excluded samples
PI-HAT	46
SEX-inconsistency	4
inbreeding coefficient	14
PCA outlier	1
Total	65

Table S3. Results of gene–based rare variant analysis

Number	Gene symbol	nVariants	*P* value	Corrected *P*	Number	Gene symbol	nVariants	*P* value	Corrected *P*
1	***INPP5J***	**2**	$\mathbf{2.99 \times 10^{-5}}$	**0.032**	534	*CDC25B*	2	0.40	1.00
2	*PLEKHG3*	3	6.57×10^{-5}	0.070	535	*TULP4*	3	0.40	1.00
3	*COL27A1*	2	2.52×10^{-4}	0.268	536	*NCOR2*	2	0.40	1.00
4	*ZFHX4*	2	3.72×10^{-4}	0.398	537	*TPO*	2	0.40	1.00
5	*TTN*	41	1.01×10^{-3}	1.00	538	*TRPM4*	2	0.40	1.00
6	*CCDC88C*	3	2.03×10^{-3}	1.00	539	*PLCG1*	2	0.40	1.00
7	*VPS52*	2	2.06×10^{-3}	1.00	540	*CHIA*	2	0.40	1.00
8	*SLC12A3*	2	2.24×10^{-3}	1.00	541	*MPO*	2	0.40	1.00
9	*VEZT*	2	2.77×10^{-3}	1.00	542	*EVC2*	3	0.40	1.00
10	*OR52E2*	2	3.47×10^{-3}	1.00	543	*HAUS6*	4	0.40	1.00
11	*WDR87*	4	5.48×10^{-3}	1.00	544	*CTNS*	2	0.40	1.00
12	*CELSR1*	4	5.56×10^{-3}	1.00	545	*CHST14*	2	0.40	1.00
13	*FBL*	2	5.95×10^{-3}	1.00	546	*SDK1*	4	0.41	1.00
14	*TBC1D16*	2	6.45×10^{-3}	1.00	547	*CSMD1*	2	0.41	1.00
15	*VPS13C*	5	6.47×10^{-3}	1.00	548	*MTMR9*	2	0.41	1.00
16	*DDX47*	2	7.73×10^{-3}	1.00	549	*FN1*	3	0.41	1.00
17	*ATP13A3*	2	8.61×10^{-3}	1.00	550	*SMAP1*	2	0.41	1.00
18	*LRRK1*	3	8.80×10^{-3}	1.00	551	*GBX1*	2	0.41	1.00
19	*MUC5B*	3	9.32×10^{-3}	1.00	552	*FSCN3*	2	0.42	1.00
20	*KIF7*	3	9.71×10^{-3}	1.00	553	*ZFHX3*	4	0.42	1.00
21	*CC2D2B*	4	0.011	1.00	554	*ZNF572*	2	0.42	1.00
22	*CHST12*	3	0.012	1.00	555	*NCR3*	2	0.42	1.00

23	*ZC3HC1*	2	0.012	1.00	556	*CASZ1*	3	0.42	1.00
24	*MAGI2*	3	0.012	1.00	557	*PDLIM2*	2	0.42	1.00
25	*SLC17A9*	2	0.012	1.00	558	*NTN3*	2	0.42	1.00
26	*TSEN54*	2	0.013	1.00	559	*WDR59*	3	0.42	1.00
27	*CENPE*	3	0.014	1.00	560	*SEMA5B*	4	0.43	1.00
28	*LDHAL6A*	2	0.014	1.00	561	*KIF13A*	2	0.43	1.00
29	*CRTAC1*	2	0.014	1.00	562	*IP6K3*	2	0.43	1.00
30	*MAN2B2*	3	0.015	1.00	563	*ZNF225*	3	0.43	1.00
31	*MYH4*	2	0.015	1.00	564	*LINGO4*	2	0.43	1.00
32	*SUCLG2*	2	0.016	1.00	565	*ARCN1*	2	0.43	1.00
33	*ARHGEF40*	4	0.016	1.00	566	*NAV1*	2	0.43	1.00
34	*GLP2R*	2	0.017	1.00	567	*GATA4*	2	0.43	1.00
35	*ACTL8*	2	0.017	1.00	568	*TMPRSS7*	3	0.43	1.00
36	*GPATCH8*	2	0.018	1.00	569	*PCDHA6*	2	0.43	1.00
37	*KRT28*	2	0.018	1.00	570	*NCOR1*	2	0.43	1.00
38	*AKAP7*	2	0.019	1.00	571	*COL20A1*	3	0.43	1.00
39	*GPC1*	2	0.020	1.00	572	*CNGB3*	2	0.43	1.00
40	*FRMD4B*	2	0.021	1.00	573	*TNK2*	2	0.44	1.00
41	*SYTL1*	2	0.021	1.00	574	*CTSW*	2	0.44	1.00
42	*LRRIQ1*	2	0.022	1.00	575	*TMEM104*	2	0.44	1.00
43	*SYCP1*	2	0.022	1.00	576	*INCENP*	2	0.44	1.00
44	*CRIM1*	3	0.022	1.00	577	*SIRPA*	2	0.44	1.00
45	*PLCB2*	2	0.022	1.00	578	*PPP2R1B*	2	0.44	1.00
46	*RPH3AL*	2	0.023	1.00	579	*GFM2*	5	0.44	1.00

47	*FLNC*	4	0.023	1.00	580	*EPB41L1*	2	0.45	1.00
48	*FRZB*	3	0.024	1.00	581	*TLN1*	2	0.45	1.00
49	*GPSM2*	2	0.024	1.00	582	*OR13C9*	2	0.45	1.00
50	*TMPRSS5*	2	0.024	1.00	583	*ATP6V0D2*	2	0.45	1.00
51	*COL28A1*	4	0.024	1.00	584	*UBE4B*	2	0.45	1.00
52	*SLC2A5*	2	0.026	1.00	585	*IGFN1*	3	0.45	1.00
53	*SIPA1L2*	2	0.030	1.00	586	*CPZ*	2	0.45	1.00
54	*SYNE2*	5	0.030	1.00	587	*ATG2B*	2	0.45	1.00
55	*NT5C1B-RDH14*	2	0.030	1.00	588	*INO80*	2	0.45	1.00
56	*ARHGEF37*	2	0.030	1.00	589	*SCN9A*	3	0.45	1.00
57	*NT5C1B*	2	0.031	1.00	590	*RAPGEF1*	2	0.45	1.00
58	*SBF2*	2	0.032	1.00	591	*ROR2*	4	0.47	1.00
59	*NSUN7*	2	0.032	1.00	592	*FCN1*	2	0.47	1.00
60	*PHRF1*	2	0.033	1.00	593	*EMILIN2*	2	0.47	1.00
61	*MYO18B*	2	0.033	1.00	594	*COL9A2*	2	0.47	1.00
62	*FAM186B*	2	0.033	1.00	595	*INSRR*	2	0.47	1.00
63	*PKD2*	2	0.034	1.00	596	*COL12A1*	2	0.47	1.00
64	*TTC16*	3	0.035	1.00	597	*HSD17B4*	2	0.47	1.00
65	*ECD*	2	0.035	1.00	598	*EXOC7*	2	0.47	1.00
66	*SDK2*	5	0.036	1.00	599	*HECTD3*	3	0.48	1.00
67	*CAND2*	4	0.037	1.00	600	*GRM6*	2	0.48	1.00
68	*C8A*	2	0.038	1.00	601	*AHI1*	2	0.48	1.00
69	*CCDC141*	2	0.038	1.00	602	*NRG2*	2	0.48	1.00
70	*CNTN6*	2	0.038	1.00	603	*SLC44A1*	2	0.48	1.00

71	*KNTC1*	5	0.038	1.00
72	*KIAA2026*	3	0.039	1.00
73	*LOXL4*	3	0.039	1.00
74	*MED13L*	2	0.040	1.00
75	*LAMA5*	3	0.041	1.00
76	*LAMA4*	3	0.042	1.00
77	*DGKI*	2	0.042	1.00
78	*CCDC116*	2	0.044	1.00
79	*IFT122*	2	0.046	1.00
80	*MACC1*	2	0.046	1.00
81	*COQ5*	2	0.046	1.00
82	*FBRS*	2	0.047	1.00
83	*SNX21*	2	0.048	1.00
84	*SLC27A1*	3	0.048	1.00
85	*OBSCN*	13	0.050	1.00
86	*MYADML2*	2	0.051	1.00
87	*LCOR*	3	0.052	1.00
88	*OTOP3*	3	0.053	1.00
89	*PPEF2*	2	0.053	1.00
90	*EYA1*	2	0.054	1.00
91	*ENDOU*	2	0.055	1.00
92	*KRI1*	2	0.055	1.00
93	*ANO4*	3	0.056	1.00
94	*PDZD3*	4	0.057	1.00
604	*PDZD2*	2	0.48	1.00
605	*ABCC12*	2	0.48	1.00
606	*KCNH6*	2	0.48	1.00
607	*CAMTA1*	3	0.48	1.00
608	*MCPH1*	2	0.48	1.00
609	*ACSF3*	2	0.48	1.00
610	*FGL1*	2	0.49	1.00
611	*VAV2*	2	0.49	1.00
612	*ADRA2B*	2	0.49	1.00
613	*HTR1A*	2	0.49	1.00
614	*MCM8*	4	0.49	1.00
615	*SLC10A7*	2	0.49	1.00
616	*DNMBP*	5	0.49	1.00
617	*DDX54*	2	0.49	1.00
618	*AGRN*	4	0.49	1.00
619	*ARID3B*	2	0.50	1.00
620	*PTPRT*	2	0.50	1.00
621	*ADAMTSL1*	7	0.50	1.00
622	*CNDP2*	2	0.50	1.00
623	*EXT2*	2	0.50	1.00
624	*MYOF*	6	0.50	1.00
625	*TNS1*	3	0.50	1.00
626	*IGSF10*	3	0.50	1.00
627	*SNCAIP*	2	0.50	1.00

95	*CNOT10*	2	0.057	1.00	628	*SEC24D*	3	0.51	1.00
96	*SVIL*	3	0.057	1.00	629	*PRPH*	2	0.51	1.00
97	*KRT82*	2	0.058	1.00	630	*NPAT*	2	0.51	1.00
98	*DOCK10*	2	0.058	1.00	631	*EML3*	2	0.51	1.00
99	*KLF12*	2	0.058	1.00	632	*FBLN7*	2	0.51	1.00
100	*FRAS1*	3	0.060	1.00	633	*AHNAK*	6	0.51	1.00
101	*LRRC9*	2	0.060	1.00	634	*WDFY4*	4	0.51	1.00
102	*ZNF592*	2	0.061	1.00	635	*PCDHB10*	2	0.51	1.00
103	*WRN*	2	0.063	1.00	636	*CAMSAP3*	2	0.51	1.00
104	*ELMO3*	2	0.066	1.00	637	*PREP*	2	0.51	1.00
105	*SULT1C4*	2	0.067	1.00	638	*MYOM2*	3	0.52	1.00
106	*AP4B1*	2	0.068	1.00	639	*RAVER1*	2	0.52	1.00
107	*HEATR1*	3	0.068	1.00	640	*FAT3*	5	0.52	1.00
108	*AKAP6*	3	0.068	1.00	641	*ARHGEF33*	2	0.52	1.00
109	*FOXM1*	2	0.068	1.00	642	*USH2A*	3	0.52	1.00
110	*IQGAP3*	2	0.070	1.00	643	*MTUS1*	2	0.52	1.00
111	*RYR2*	2	0.070	1.00	644	*UBR4*	2	0.52	1.00
112	*DNAI1*	2	0.072	1.00	645	*ZC3H4*	2	0.52	1.00
113	*SLC35A1*	2	0.073	1.00	646	*HS3ST3B1*	2	0.53	1.00
114	*BNC1*	2	0.073	1.00	647	*UHRF1BP1*	4	0.53	1.00
115	*ITIH5*	2	0.074	1.00	648	*TNXB*	6	0.53	1.00
116	*SRRM2*	4	0.074	1.00	649	*CORIN*	2	0.53	1.00
117	*SLC12A7*	2	0.075	1.00	650	*RNF148*	2	0.53	1.00
118	*PDK2*	2	0.077	1.00	651	*MYH6*	5	0.53	1.00

119	*NRP1*	2	0.077	1.00	652	*MYO1D*	2	0.53	1.00
120	*PLEC*	9	0.078	1.00	653	*BCAM*	2	0.53	1.00
121	*AKAP13*	2	0.079	1.00	654	*RAPH1*	3	0.53	1.00
122	*RGL2*	2	0.080	1.00	655	*PEG3*	2	0.53	1.00
123	*ARHGAP44*	2	0.082	1.00	656	*MICAL1*	6	0.53	1.00
124	*JPH2*	2	0.082	1.00	657	*KRTAP6-3*	2	0.53	1.00
125	*WWOX*	2	0.082	1.00	658	*PLA2G7*	3	0.53	1.00
126	*ATP9B*	3	0.082	1.00	659	*DUPD1*	3	0.54	1.00
127	*EMILIN3*	2	0.082	1.00	660	*XIRP2*	6	0.54	1.00
128	*DNAH6*	3	0.083	1.00	661	*SEMA3G*	2	0.54	1.00
129	*FLYWCH1*	2	0.083	1.00	662	*BAZ2A*	2	0.54	1.00
130	*DDX49*	3	0.084	1.00	663	*DCHS1*	3	0.54	1.00
131	*ZNF609*	2	0.084	1.00	664	*FAT1*	6	0.54	1.00
132	*EPOR*	2	0.085	1.00	665	*HIVEP1*	2	0.54	1.00
133	*ABCA7*	3	0.085	1.00	666	*ALDH1L1*	2	0.54	1.00
134	*GC*	2	0.086	1.00	667	*USP53*	2	0.54	1.00
135	*RNF123*	3	0.086	1.00	668	*EML2*	3	0.54	1.00
136	*ERBB3*	3	0.088	1.00	669	*ZNF816*	3	0.54	1.00
137	*ZNF469*	2	0.088	1.00	670	*PDE11A*	2	0.55	1.00
138	*PREPL*	3	0.088	1.00	671	*SORBS2*	2	0.55	1.00
139	*COL5A3*	2	0.088	1.00	672	*ZNF646*	2	0.55	1.00
140	*BMS1*	3	0.089	1.00	673	*FREM3*	2	0.55	1.00
141	*SULF2*	2	0.089	1.00	674	*ITGA7*	2	0.55	1.00
142	*RAI14*	2	0.089	1.00	675	*CLCA4*	3	0.55	1.00

143	*ATR*	2	0.090	1.00	676	*CCT6B*	2	0.55	1.00
144	*APC*	2	0.090	1.00	677	*RGS14*	2	0.55	1.00
145	*UBR1*	3	0.090	1.00	678	*ANK1*	3	0.55	1.00
146	*PIK3C2B*	3	0.091	1.00	679	*AP1G1*	2	0.56	1.00
147	*SV2B*	2	0.092	1.00	680	*ILVBL*	2	0.56	1.00
148	*DNAH8*	6	0.093	1.00	681	*MON2*	2	0.56	1.00
149	*YTHDC2*	2	0.093	1.00	682	*NUP210L*	3	0.56	1.00
150	*BCHE*	2	0.095	1.00	683	*DNAH3*	4	0.56	1.00
151	*UNC13C*	3	0.095	1.00	684	*TLR5*	2	0.57	1.00
152	*LRBA*	4	0.097	1.00	685	*ADAMTS7*	2	0.57	1.00
153	*ACAD10*	2	0.097	1.00	686	*DUOX2*	3	0.57	1.00
154	*METTL21C*	2	0.100	1.00	687	*ITPRIP*	2	0.57	1.00
155	*CACNA1A*	4	0.100	1.00	688	*RAB3GAP2*	3	0.57	1.00
156	*FAM189B*	2	0.101	1.00	689	*LNPEP*	2	0.57	1.00
157	*KDM3B*	2	0.101	1.00	690	*KLHL30*	2	0.57	1.00
158	*OTOG*	4	0.101	1.00	691	*TRUB1*	2	0.57	1.00
159	*NEB*	11	0.102	1.00	692	*SHKBP1*	2	0.58	1.00
160	*F5*	2	0.102	1.00	693	*OTOF*	2	0.58	1.00
161	*FARP1*	2	0.104	1.00	694	*TLR6*	2	0.58	1.00
162	*ALKBH2*	3	0.104	1.00	695	*ST7L*	2	0.58	1.00
163	*CDK13*	3	0.104	1.00	696	*GAD1*	2	0.58	1.00
164	*MUC4*	2	0.106	1.00	697	*C4orf47*	2	0.58	1.00
165	*LAMB3*	3	0.108	1.00	698	*PFAS*	2	0.58	1.00
166	*PIEZO1*	3	0.108	1.00	699	*INTS1*	2	0.58	1.00

167	*GOT2*	2	0.110	1.00	700	*CFTR*	3	0.58	1.00
168	*SLC10A1*	3	0.111	1.00	701	*SLC38A6*	2	0.59	1.00
169	*GANAB*	2	0.111	1.00	702	*CHRNB3*	2	0.59	1.00
170	*NACAD*	4	0.111	1.00	703	*DNAH5*	5	0.59	1.00
171	*TGM6*	5	0.112	1.00	704	*KIAA0319*	2	0.59	1.00
172	*HOXC13*	2	0.113	1.00	705	*VAX2*	2	0.59	1.00
173	*TGM1*	3	0.113	1.00	706	*PLD1*	2	0.59	1.00
174	*KRT77*	2	0.114	1.00	707	*PRDM2*	2	0.59	1.00
175	*BPIFC*	3	0.114	1.00	708	*HGD*	2	0.60	1.00
176	*NCLN*	3	0.117	1.00	709	*LAMA3*	2	0.60	1.00
177	*FRMPD1*	2	0.117	1.00	710	*NOP14*	2	0.60	1.00
178	*RASAL2*	3	0.117	1.00	711	*MIIP*	2	0.60	1.00
179	*ZNF574*	2	0.118	1.00	712	*SRRM3*	2	0.60	1.00
180	*GNE*	2	0.119	1.00	713	*LDB1*	2	0.60	1.00
181	*CHST5*	2	0.120	1.00	714	*OCM2*	2	0.60	1.00
182	*ALCAM*	3	0.121	1.00	715	*PCDH15*	3	0.60	1.00
183	*DNHD1*	2	0.123	1.00	716	*PIEZO2*	2	0.60	1.00
184	*PAAF1*	2	0.123	1.00	717	*SACS*	3	0.60	1.00
185	*TOP1MT*	3	0.123	1.00	718	*MDGA1*	2	0.60	1.00
186	*CACNA1H*	4	0.123	1.00	719	*DARS2*	2	0.60	1.00
187	*PP2D1*	4	0.124	1.00	720	*FBN2*	2	0.60	1.00
188	*COL2A1*	2	0.125	1.00	721	*CMYA5*	2	0.61	1.00
189	*ATG4B*	2	0.125	1.00	722	*TBC1D2*	2	0.61	1.00
190	*PLOD2*	3	0.125	1.00	723	*FGGY*	3	0.61	1.00

191	*SPEF2*	3	0.125	1.00
192	*KRT6A*	2	0.127	1.00
193	*KIF25*	2	0.13	1.00
194	*XPOT*	2	0.13	1.00
195	*KIAA1841*	2	0.13	1.00
196	*CLTCL1*	3	0.13	1.00
197	*B3GALT1*	2	0.13	1.00
198	*ANGPTL6*	2	0.13	1.00
199	*FNDC3B*	2	0.13	1.00
200	*DNAJB8*	3	0.13	1.00
201	*RDH16*	2	0.13	1.00
202	*FOXR1*	2	0.13	1.00
203	*MYO1F*	3	0.13	1.00
204	*SLC15A5*	3	0.14	1.00
205	*FMN1*	3	0.14	1.00
206	*LIPJ*	2	0.14	1.00
207	*KIF21A*	2	0.14	1.00
208	*TIMELESS*	2	0.14	1.00
209	*TARSL2*	2	0.14	1.00
210	*CROCC*	3	0.14	1.00
211	*RBM34*	2	0.14	1.00
212	*LGR5*	2	0.14	1.00
213	*FGFR1*	2	0.14	1.00
214	*KRT2*	3	0.15	1.00
724	*NUMA1*	3	0.61	1.00
725	*SCARA3*	2	0.61	1.00
726	*GAPDHS*	2	0.61	1.00
727	*MAGEL2*	2	0.61	1.00
728	*CPOX*	2	0.62	1.00
729	*RB1CC1*	2	0.62	1.00
730	*SHANK2*	2	0.62	1.00
731	*CALD1*	2	0.62	1.00
732	*UTP14C*	2	0.63	1.00
733	*IL17RD*	2	0.63	1.00
734	*LAMA2*	4	0.63	1.00
735	*PRKAG2*	2	0.63	1.00
736	*ISM1*	2	0.63	1.00
737	*SAMD9L*	2	0.63	1.00
738	*URB1*	2	0.63	1.00
739	*CPNE5*	3	0.64	1.00
740	*CCDC125*	2	0.64	1.00
741	*DLG5*	2	0.64	1.00
742	*PTGIR*	2	0.64	1.00
743	*STIM1*	2	0.64	1.00
744	*MCM10*	2	0.64	1.00
745	*FAM114A1*	2	0.64	1.00
746	*ARHGAP10*	2	0.65	1.00
747	*CYP26C1*	2	0.65	1.00

215	*SFT2D3*	2	0.15	1.00	748	*VPS13A*	2	0.65	1.00
216	*ROS1*	2	0.15	1.00	749	*SLC25A21*	2	0.65	1.00
217	*ARSK*	2	0.15	1.00	750	*CUBN*	4	0.65	1.00
218	*RAPGEF4*	3	0.15	1.00	751	*CARD9*	2	0.65	1.00
219	*NCOA2*	2	0.15	1.00	752	*OPLAH*	4	0.65	1.00
220	*OR5AU1*	2	0.15	1.00	753	*NEDD4*	3	0.65	1.00
221	*CUL9*	3	0.15	1.00	754	*UGT1A5*	2	0.65	1.00
222	*FAM166A*	2	0.15	1.00	755	*OR5K3*	2	0.65	1.00
223	*ALG3*	2	0.15	1.00	756	*HERC1*	2	0.65	1.00
224	*ITGA3*	3	0.15	1.00	757	*UNC80*	3	0.65	1.00
225	*ADAMTS18*	2	0.15	1.00	758	*CRISP1*	2	0.66	1.00
226	*PIGG*	2	0.15	1.00	759	*CPPED1*	2	0.66	1.00
227	*THADA*	3	0.16	1.00	760	*BFSP2*	2	0.66	1.00
228	*AKAP9*	3	0.16	1.00	761	*CEP350*	2	0.66	1.00
229	*PEAK1*	2	0.16	1.00	762	*MTMR4*	2	0.66	1.00
230	*DYNC2H1*	3	0.16	1.00	763	*FAM83H*	2	0.67	1.00
231	*CD109*	4	0.16	1.00	764	*ATF7IP2*	3	0.67	1.00
232	*CEP290*	2	0.16	1.00	765	*ARID1B*	2	0.67	1.00
233	*COBLL1*	2	0.16	1.00	766	*MYRIP*	2	0.67	1.00
234	*BIVM-ERCC5*	2	0.16	1.00	767	*FCRL1*	2	0.68	1.00
235	*ZNF226*	2	0.16	1.00	768	*PDE3A*	2	0.68	1.00
236	*RNF17*	2	0.16	1.00	769	*MAP3K1*	2	0.68	1.00
237	*TNNI3K*	2	0.16	1.00	770	*MACF1*	6	0.68	1.00
238	*CYP2A7*	2	0.16	1.00	771	*ARVCF*	2	0.68	1.00

239	*ANXA10*	2	0.17	1.00	772	*SORBS1*	4	0.68	1.00
240	*APLP2*	2	0.17	1.00	773	*CRYGA*	3	0.69	1.00
241	*FPGT-TNNI3K*	2	0.17	1.00	774	*DICER1*	2	0.69	1.00
242	*FAM184B*	2	0.17	1.00	775	*DMXL1*	2	0.69	1.00
243	*PKHD1*	3	0.17	1.00	776	*WDR31*	2	0.69	1.00
244	*TNC*	2	0.17	1.00	777	*LRCH3*	2	0.69	1.00
245	*TRIP11*	4	0.17	1.00	778	*ITGA9*	2	0.69	1.00
246	*AMPD1*	2	0.17	1.00	779	*ILDR1*	2	0.69	1.00
247	*CIT*	2	0.17	1.00	780	*OSBPL6*	2	0.70	1.00
248	*WDFY3*	2	0.17	1.00	781	*C7orf50*	2	0.70	1.00
249	*FAM149A*	2	0.17	1.00	782	*UTRN*	4	0.70	1.00
250	*LAMA1*	2	0.17	1.00	783	*HPS3*	2	0.70	1.00
251	*SLC16A13*	2	0.18	1.00	784	*KLHL24*	2	0.71	1.00
252	*CNTN5*	2	0.18	1.00	785	*ABCC1*	5	0.71	1.00
253	*GPLD1*	2	0.18	1.00	786	*SMG1*	3	0.71	1.00
254	*NCKAP1L*	2	0.18	1.00	787	*FAM161B*	3	0.71	1.00
255	*ARMC2*	2	0.18	1.00	788	*UBA7*	3	0.71	1.00
256	*DNAH9*	4	0.18	1.00	789	*CA1*	2	0.72	1.00
257	*AVIL*	4	0.18	1.00	790	*SETD2*	2	0.72	1.00
258	*PCDHGA7*	4	0.18	1.00	791	*PCDHGB6*	2	0.72	1.00
259	*VWA3A*	2	0.18	1.00	792	*LMO7*	2	0.72	1.00
260	*C2CD3*	2	0.18	1.00	793	*PDZD7*	2	0.72	1.00
261	*ZNF502*	2	0.18	1.00	794	*ACACB*	4	0.72	1.00
262	*PM20D1*	2	0.18	1.00	795	*SLC12A4*	2	0.72	1.00

263	*ANKAR*	2	0.18	1.00	796	*DNAH7*	2	0.73	1.00
264	*COL6A2*	3	0.18	1.00	797	*PPL*	2	0.73	1.00
265	*SH3TC1*	2	0.18	1.00	798	*COL4A2*	4	0.73	1.00
266	*SIPA1L3*	3	0.19	1.00	799	*TTC6*	3	0.73	1.00
267	*PKD1*	4	0.19	1.00	800	*GABRA6*	3	0.73	1.00
268	*MYOG*	2	0.19	1.00	801	*SPO11*	2	0.74	1.00
269	*FCRL5*	2	0.19	1.00	802	*ROBO4*	2	0.74	1.00
270	*CTSA*	2	0.19	1.00	803	*DNAH2*	6	0.74	1.00
271	*CDH23*	2	0.19	1.00	804	*VAT1L*	3	0.74	1.00
272	*SLC28A2*	2	0.19	1.00	805	*MYH7B*	4	0.74	1.00
273	*PLIN1*	2	0.19	1.00	806	*DPH1*	2	0.74	1.00
274	*CTSH*	2	0.19	1.00	807	*PDS5A*	2	0.74	1.00
275	*CWF19L2*	2	0.19	1.00	808	*MYH1*	3	0.74	1.00
276	*RGS3*	2	0.19	1.00	809	*ZNF638*	2	0.74	1.00
277	*COL15A1*	2	0.19	1.00	810	*ALDH9A1*	2	0.75	1.00
278	*ATF6B*	2	0.19	1.00	811	*POPDC2*	2	0.75	1.00
279	*SPTBN5*	2	0.19	1.00	812	*VWA2*	2	0.75	1.00
280	*HYOU1*	2	0.19	1.00	813	*PDE6A*	2	0.75	1.00
281	*SRCAP*	2	0.19	1.00	814	*SLC38A7*	3	0.75	1.00
282	*CILP*	4	0.19	1.00	815	*FIGN*	2	0.75	1.00
283	*CGN*	3	0.19	1.00	816	*SCN10A*	3	0.76	1.00
284	*UNC79*	2	0.19	1.00	817	*LRRC66*	2	0.76	1.00
285	*MTMR11*	3	0.19	1.00	818	*SLC9A9*	2	0.76	1.00
286	*CLIP4*	2	0.20	1.00	819	*CLCN1*	2	0.76	1.00

287	*CES5A*	2	0.20	1.00	820	*ESPL1*	2	0.76	1.00
288	*GAS2L2*	2	0.20	1.00	821	*INSC*	2	0.76	1.00
289	*HMCN1*	4	0.20	1.00	822	*NINL*	3	0.76	1.00
290	*GGT5*	2	0.20	1.00	823	*TELO2*	2	0.76	1.00
291	*GRIN2C*	2	0.20	1.00	824	*LTB4R2*	2	0.77	1.00
292	*BOC*	2	0.20	1.00	825	*MAP7D1*	3	0.77	1.00
293	*SLC5A4*	3	0.20	1.00	826	*ACAN*	5	0.77	1.00
294	*HLTF*	3	0.20	1.00	827	*DCHS2*	5	0.77	1.00
295	*IARS2*	2	0.20	1.00	828	*MST1R*	2	0.77	1.00
296	*CEP164*	2	0.20	1.00	829	*ASTN2*	3	0.77	1.00
297	*PHLDB1*	2	0.20	1.00	830	*ZNF534*	2	0.78	1.00
298	*ERCC5*	2	0.20	1.00	831	*PER3*	2	0.78	1.00
299	*HLA-C*	2	0.20	1.00	832	*FOXD4*	2	0.78	1.00
300	*FSCN2*	3	0.20	1.00	833	*DNAH1*	4	0.78	1.00
301	*COL6A6*	3	0.20	1.00	834	*MDN1*	4	0.78	1.00
302	*SYTL3*	2	0.20	1.00	835	*MAN1B1*	2	0.78	1.00
303	*KAT2B*	2	0.21	1.00	836	*HMGXB3*	2	0.79	1.00
304	*LDB3*	2	0.21	1.00	837	*ARHGEF17*	3	0.79	1.00
305	*PRDM16*	2	0.21	1.00	838	*PPIC*	3	0.79	1.00
306	*MSH6*	3	0.21	1.00	839	*SLC7A2*	3	0.79	1.00
307	*LRP2*	3	0.21	1.00	840	*MLX*	3	0.79	1.00
308	*ARHGAP33*	2	0.21	1.00	841	*SLC47A1*	3	0.79	1.00
309	*KIF26B*	2	0.21	1.00	842	*JMJD7-PLA2G4B*	3	0.80	1.00
310	*C1QTNF2*	2	0.21	1.00	843	*PLA2G4B*	3	0.80	1.00

311	*PKHD1L1*	6	0.21	1.00	844	*FANCM*	2	0.80	1.00
312	*ENPP2*	2	0.22	1.00	845	*ADH7*	2	0.80	1.00
313	*DCC*	4	0.22	1.00	846	*ABI3*	2	0.80	1.00
314	*KLHL10*	2	0.22	1.00	847	*LRP1B*	2	0.80	1.00
315	*PIK3C2G*	2	0.22	1.00	848	*BRCA1*	3	0.81	1.00
316	*PLEKHH3*	2	0.22	1.00	849	*NID1*	5	0.81	1.00
317	*RAMP3*	2	0.22	1.00	850	*MAN2A1*	2	0.81	1.00
318	*BFAR*	2	0.22	1.00	851	*SLC27A3*	3	0.81	1.00
319	*OLFML2A*	2	0.22	1.00	852	*CD101*	2	0.82	1.00
320	*LRRC15*	2	0.22	1.00	853	*EFCAB6*	2	0.82	1.00
321	*GSC2*	2	0.22	1.00	854	*CSPP1*	2	0.83	1.00
322	*ABCC4*	2	0.22	1.00	855	*KDM4C*	2	0.83	1.00
323	*ADAMTS16*	2	0.22	1.00	856	*GABRP*	2	0.83	1.00
324	*STARD5*	2	0.22	1.00	857	*CD36*	2	0.83	1.00
325	*GCAT*	2	0.23	1.00	858	*POLR3F*	2	0.84	1.00
326	*LRRIQ3*	2	0.23	1.00	859	*NBEAL2*	2	0.84	1.00
327	*XPO7*	2	0.23	1.00	860	*DMGDH*	3	0.84	1.00
328	*ARHGAP9*	2	0.23	1.00	861	*CLSTN3*	2	0.84	1.00
329	*RYR1*	2	0.23	1.00	862	*CHCHD5*	2	0.84	1.00
330	*PTCD1*	2	0.23	1.00	863	*CCT5*	2	0.85	1.00
331	*COL24A1*	4	0.23	1.00	864	*TMEM161A*	2	0.85	1.00
332	*PCNT*	3	0.23	1.00	865	*ZNF772*	2	0.85	1.00
333	*ENPP1*	2	0.23	1.00	866	*IGSF9B*	4	0.86	1.00
334	*ANK2*	4	0.23	1.00	867	*CDHR1*	2	0.86	1.00

335	*ST5*	2	0.23	1.00	868	*MORN1*	2	0.86	1.00
336	*CROT*	2	0.24	1.00	869	*LAMB1*	4	0.86	1.00
337	*PCDHGA5*	2	0.24	1.00	870	*PTPRO*	2	0.86	1.00
338	*COL22A1*	2	0.24	1.00	871	*TRPM6*	3	0.86	1.00
339	*NAV2*	3	0.24	1.00	872	*NSMCE2*	2	0.87	1.00
340	*ADAMTS14*	3	0.24	1.00	873	*HSPG2*	6	0.88	1.00
341	*MTFMT*	3	0.24	1.00	874	*ABCC11*	3	0.88	1.00
342	*MYOM3*	3	0.24	1.00	875	*THNSL2*	3	0.88	1.00
343	*HLA-DMA*	2	0.24	1.00	876	*ADAMTSL4*	3	0.89	1.00
344	*KIF3C*	2	0.24	1.00	877	*TEP1*	3	0.89	1.00
345	*ABCC8*	2	0.24	1.00	878	*KIF6*	3	0.89	1.00
346	*PRDM1*	2	0.24	1.00	879	*REM1*	3	0.89	1.00
347	*MTRF1L*	2	0.24	1.00	880	*ABCB5*	3	1.00	1.00
348	*KIF27*	2	0.24	1.00	881	*ABLIM3*	2	1.00	1.00
349	*FOXA2*	3	0.25	1.00	882	*ADAM7*	2	1.00	1.00
350	*GCKR*	2	0.25	1.00	883	*ADAMTS1*	2	1.00	1.00
351	*ADAMTS9*	2	0.25	1.00	884	*ATP10B*	2	1.00	1.00
352	*TJP2*	2	0.25	1.00	885	*SPTA1*	3	1.00	1.00
353	*SPATS2*	2	0.25	1.00	886	*ASTN1*	2	1.00	1.00
354	*FSIP2*	3	0.25	1.00	887	*CADPS*	2	1.00	1.00
355	*NUB1*	2	0.25	1.00	888	*CCDC61*	2	1.00	1.00
356	*KLHDC2*	2	0.25	1.00	889	*CLK3*	2	1.00	1.00
357	*BMP2*	2	0.25	1.00	890	*CNTROB*	3	1.00	1.00
358	*FHOD1*	3	0.25	1.00	891	*SIGLEC15*	2	1.00	1.00

359	*RUFY2*	2	0.25	1.00
360	*SYNE1*	4	0.25	1.00
361	*PDIA5*	2	0.25	1.00
362	*ABCA10*	2	0.25	1.00
363	*TMC2*	2	0.25	1.00
364	*RARRES1*	2	0.25	1.00
365	*IGHMBP2*	2	0.26	1.00
366	*LRRFIP2*	2	0.26	1.00
367	*MYO7B*	3	0.26	1.00
368	*MYO10*	2	0.26	1.00
369	*GAA*	2	0.26	1.00
370	*FAM151A*	3	0.26	1.00
371	*IGDCC3*	4	0.26	1.00
372	*SLCO1A2*	3	0.26	1.00
373	*FLNB*	5	0.26	1.00
374	*FZD4*	2	0.26	1.00
375	*ADAMTS5*	2	0.27	1.00
376	*CLOCK*	2	0.27	1.00
377	*RANBP6*	2	0.27	1.00
378	*ANKHD1-EIF4EBP3*	2	0.27	1.00
379	*KIRREL2*	3	0.27	1.00
380	*ATRN*	2	0.27	1.00
381	*GRIN2D*	2	0.27	1.00
382	*TTC7A*	3	0.27	1.00
892	*TMEM54*	2	1.00	1.00
893	*TPR*	2	1.00	1.00
894	*ATP8B2*	2	1.00	1.00
895	*NEK3*	2	1.00	1.00
896	*ZP4*	2	1.00	1.00
897	*CEACAM16*	2	1.00	1.00
898	*DBF4B*	2	1.00	1.00
899	*DMBT1*	4	1.00	1.00
900	*CEP72*	2	1.00	1.00
901	*DNAH17*	7	1.00	1.00
902	*MICALL1*	2	1.00	1.00
903	*COG5*	2	1.00	1.00
904	*COL4A4*	2	1.00	1.00
905	*CUL7*	2	1.00	1.00
906	*CYP2A13*	2	1.00	1.00
907	*FAH*	2	1.00	1.00
908	*CC2D1B*	4	1.00	1.00
909	*LTBP2*	2	1.00	1.00
910	*DSCAML1*	2	1.00	1.00
911	*ITGA8*	2	1.00	1.00
912	*DCBLD1*	2	1.00	1.00
913	*ENO3*	2	1.00	1.00
914	*HAPLN3*	2	1.00	1.00
915	*CYP26B1*	2	1.00	1.00

383	*MAP1B*	2	0.27	1.00	916	*ELMOD1*	2	1.00	1.00
384	*CDH1*	2	0.27	1.00	917	*NFATC4*	2	1.00	1.00
385	*TMEM82*	2	0.27	1.00	918	*ABCA5*	2	1.00	1.00
386	*LRRC46*	2	0.27	1.00	919	*ALDH1B1*	2	1.00	1.00
387	*ADAMTS12*	2	0.27	1.00	920	*DSP*	3	1.00	1.00
388	*KIAA0556*	3	0.28	1.00	921	*FDXR*	2	1.00	1.00
389	*RNPEP*	2	0.28	1.00	922	*KCNV2*	2	1.00	1.00
390	*PPWD1*	2	0.28	1.00	923	*UPB1*	2	1.00	1.00
391	*NOL10*	2	0.28	1.00	924	*FOXN1*	2	1.00	1.00
392	*PACRGL*	2	0.28	1.00	925	*PANK2*	2	1.00	1.00
393	*EPB41L4B*	2	0.28	1.00	926	*ENTPD3*	2	1.00	1.00
394	*RXFP3*	2	0.28	1.00	927	*FAM200A*	2	1.00	1.00
395	*STON1-GTF2A1L*	2	0.28	1.00	928	*FOXQ1*	2	1.00	1.00
396	*MTOR*	2	0.28	1.00	929	*CLCN6*	2	1.00	1.00
397	*OR9K2*	2	0.28	1.00	930	*GALNT10*	2	1.00	1.00
398	*ANKHD1*	2	0.28	1.00	931	*CNTN2*	2	1.00	1.00
399	*SHE*	2	0.28	1.00	932	*IGFALS*	2	1.00	1.00
400	*COL17A1*	2	0.29	1.00	933	*FANCL*	2	1.00	1.00
401	*DSG3*	2	0.29	1.00	934	*GPR151*	3	1.00	1.00
402	*ZNF280A*	2	0.29	1.00	935	*GRIN3B*	3	1.00	1.00
403	*NUP214*	2	0.29	1.00	936	*GET4*	2	1.00	1.00
404	*DNAH14*	3	0.29	1.00	937	*GOLGA4*	3	1.00	1.00
405	*OR5T2*	2	0.29	1.00	938	*MELK*	2	1.00	1.00
406	*PRODH*	2	0.29	1.00	939	*HPX*	2	1.00	1.00

407	*PLEKHH2*	2	0.29	1.00
408	*GPR84*	2	0.29	1.00
409	*PREB*	2	0.30	1.00
410	*LRIG1*	2	0.30	1.00
411	*MYO5B*	4	0.30	1.00
412	*C7orf57*	2	0.30	1.00
413	*CLCA2*	2	0.30	1.00
414	*CHL1*	3	0.30	1.00
415	*TRIP12*	2	0.30	1.00
416	*ADCY10*	2	0.30	1.00
417	*TMEM102*	2	0.30	1.00
418	*ANKS6*	3	0.30	1.00
419	*CHRNB1*	2	0.30	1.00
420	*PTK6*	4	0.30	1.00
421	*MAMDC4*	2	0.30	1.00
422	*RELN*	2	0.30	1.00
423	*IMP4*	2	0.30	1.00
424	*NOTCH1*	2	0.30	1.00
425	*SLC18A1*	2	0.30	1.00
426	*FLG*	6	0.30	1.00
427	*SEC14L3*	2	0.31	1.00
428	*UBE4A*	2	0.31	1.00
429	*ANKLE1*	2	0.31	1.00
430	*ASPM*	3	0.31	1.00

940	*NDST4*	3	1.00	1.00
941	*NMRAL1*	2	1.00	1.00
942	*HELB*	2	1.00	1.00
943	*SIGLEC1*	4	1.00	1.00
944	*HIP1R*	2	1.00	1.00
945	*LILRB2*	4	1.00	1.00
946	*OGDHL*	2	1.00	1.00
947	*ACSM4*	2	1.00	1.00
948	*ADAMTSL3*	2	1.00	1.00
949	*BPIFB4*	2	1.00	1.00
950	*OTOP1*	2	1.00	1.00
951	*OXR1*	2	1.00	1.00
952	*MEGF8*	4	1.00	1.00
953	*PCM1*	2	1.00	1.00
954	*MAD1L1*	2	1.00	1.00
955	*SRMS*	3	1.00	1.00
956	*RPL3L*	3	1.00	1.00
957	*SEC31B*	2	1.00	1.00
958	*LRP4*	2	1.00	1.00
959	*SH3PXD2A*	2	1.00	1.00
960	*SLC12A6*	2	1.00	1.00
961	*L3MBTL3*	2	1.00	1.00
962	*NAALADL1*	2	1.00	1.00
963	*PRX*	2	1.00	1.00

431	*TSC1*	2	0.31	1.00	964	*KIDINS220*	2	1.00	1.00
432	*PSD3*	2	0.31	1.00	965	*PCDHA8*	2	1.00	1.00
433	*COL18A1*	2	0.31	1.00	966	*SLC5A11*	2	1.00	1.00
434	*DNAH10*	2	0.31	1.00	967	*LAMB2*	2	1.00	1.00
435	*WDR7*	2	0.31	1.00	968	*SLC7A6*	2	1.00	1.00
436	*TTC28*	2	0.31	1.00	969	*ADAMTS20*	2	1.00	1.00
437	*FCHO1*	2	0.31	1.00	970	*AGBL1*	2	1.00	1.00
438	*MAP4K5*	2	0.31	1.00	971	*DGKH*	2	1.00	1.00
439	*CCDC3*	2	0.31	1.00	972	*LRFN2*	2	1.00	1.00
440	*IFIH1*	3	0.31	1.00	973	*LRRK2*	3	1.00	1.00
441	*RAD52*	2	0.32	1.00	974	*PCDHGA12*	2	1.00	1.00
442	*SMO*	2	0.32	1.00	975	*UNC5B*	2	1.00	1.00
443	*EPPK1*	2	0.32	1.00	976	*MCM9*	2	1.00	1.00
444	*RBM28*	2	0.32	1.00	977	*OR10V1*	2	1.00	1.00
445	*COL11A1*	2	0.32	1.00	978	*ZNF239*	3	1.00	1.00
446	*LTK*	2	0.32	1.00	979	*LY75*	2	1.00	1.00
447	*TCHH*	2	0.32	1.00	980	*MEA1*	2	1.00	1.00
448	*PAFAH2*	2	0.32	1.00	981	*MYH8*	3	1.00	1.00
449	*TG*	3	0.32	1.00	982	*OR5M8*	2	1.00	1.00
450	*NLRX1*	4	0.32	1.00	983	*SLC7A9*	2	1.00	1.00
451	*NAGS*	2	0.32	1.00	984	*TAOK2*	4	1.00	1.00
452	*COL6A3*	5	0.32	1.00	985	*TRPM3*	2	1.00	1.00
453	*PCDHGA6*	2	0.33	1.00	986	*LY75-CD302*	2	1.00	1.00
454	*ATP10A*	5	0.33	1.00	987	*MCM2*	3	1.00	1.00

455	*MAP2*	2	0.33	1.00	988	*SSC5D*	2	1.00	1.00
456	*DOCK8*	3	0.33	1.00	989	*TBL3*	2	1.00	1.00
457	*MICAL3*	3	0.33	1.00	990	*UBA6*	3	1.00	1.00
458	*NPHP4*	2	0.33	1.00	991	*MGLL*	2	1.00	1.00
459	*CSMD2*	6	0.33	1.00	992	*MYO18A*	3	1.00	1.00
460	*PPM1J*	3	0.33	1.00	993	*TMEM143*	2	1.00	1.00
461	*SCIN*	2	0.33	1.00	994	*VPS13B*	3	1.00	1.00
462	*TBX3*	2	0.33	1.00	995	*HHIPL2*	2	1.00	1.00
463	*UBAP2*	2	0.33	1.00	996	*MPHOSPH10*	2	1.00	1.00
464	*VWF*	3	0.34	1.00	997	*NHSL1*	2	1.00	1.00
465	*CCDC78*	2	0.34	1.00	998	*TMPRSS9*	2	1.00	1.00
466	*ST18*	3	0.34	1.00	999	*HIVEP3*	4	1.00	1.00
467	*GRPEL1*	2	0.34	1.00	1000	*OTOGL*	2	1.00	1.00
468	*PRRC2A*	3	0.34	1.00	1001	*UNC13B*	2	1.00	1.00
469	*STAB1*	3	0.34	1.00	1002	*AGL*	2	1.00	1.00
470	*UROC1*	2	0.34	1.00	1003	*ALOX15B*	2	1.00	1.00
471	*CMA1*	3	0.34	1.00	1004	*APOE*	2	1.00	1.00
472	*RALGAPA1*	2	0.34	1.00	1005	*CHD7*	2	1.00	1.00
473	*SPOPL*	2	0.34	1.00	1006	*DZIP1*	2	1.00	1.00
474	*HLA-A*	2	0.34	1.00	1007	*RASA1*	2	1.00	1.00
475	*ABCA8*	3	0.34	1.00	1008	*TRRAP*	2	1.00	1.00
476	*TNRC6C*	2	0.35	1.00	1009	*PER1*	3	1.00	1.00
477	*MYO1H*	4	0.35	1.00	1010	*PCOLCE2*	2	1.00	1.00
478	*VPS13D*	2	0.35	1.00	1011	*SART1*	4	1.00	1.00

479	*PARD3*	3	0.35	1.00
480	*AK7*	2	0.35	1.00
481	*PHKB*	2	0.35	1.00
482	*GALNT8*	2	0.35	1.00
483	*USP38*	2	0.35	1.00
484	*RECQL*	2	0.35	1.00
485	*SLITRK5*	3	0.35	1.00
486	*MYO15A*	5	0.35	1.00
487	*CCDC8*	2	0.36	1.00
488	*TTC30B*	2	0.36	1.00
489	*SH3D21*	2	0.36	1.00
490	*CPA5*	2	0.36	1.00
491	*AUTS2*	2	0.36	1.00
492	*HOXA9*	2	0.36	1.00
493	*CDH5*	2	0.36	1.00
494	*CNTRL*	2	0.36	1.00
495	*EPB42*	2	0.36	1.00
496	*PLA2G4F*	2	0.36	1.00
497	*FCRL3*	3	0.36	1.00
498	*KIF9*	2	0.36	1.00
499	*SMCR8*	2	0.36	1.00
500	*KDM6B*	2	0.37	1.00
501	*KIF16B*	2	0.37	1.00
502	*DGKG*	2	0.37	1.00
1012	*PCYT1A*	2	1.00	1.00
1013	*PTPRQ*	2	1.00	1.00
1014	*SLIT3*	3	1.00	1.00
1015	*ZNF189*	2	1.00	1.00
1016	*NPPC*	2	1.00	1.00
1017	*RNF157*	2	1.00	1.00
1018	*SCUBE2*	2	1.00	1.00
1019	*ZNF573*	3	1.00	1.00
1020	*KCNC4*	2	1.00	1.00
1021	*RIMS1*	2	1.00	1.00
1022	*TTC37*	4	1.00	1.00
1023	*ZNF584*	2	1.00	1.00
1024	*OBSL1*	3	1.00	1.00
1025	*SASH1*	2	1.00	1.00
1026	*ALOXE3*	2	1.00	1.00
1027	*C11orf49*	2	1.00	1.00
1028	*CHMP7*	2	1.00	1.00
1029	*MCM3AP*	2	1.00	1.00
1030	*LRRC71*	2	1.00	1.00
1031	*STAB2*	2	1.00	1.00
1032	*PLB1*	2	1.00	1.00
1033	*ROBO1*	2	1.00	1.00
1034	*LUZP1*	2	1.00	1.00
1035	*TBKBP1*	2	1.00	1.00

503	*FRYL*	2	0.37	1.00
504	*SMYD1*	2	0.37	1.00
505	*MAP2K3*	2	0.37	1.00
506	*DNAH11*	8	0.37	1.00
507	*IL17F*	2	0.37	1.00
508	*NBN*	2	0.37	1.00
509	*FANCI*	2	0.38	1.00
510	*PTGIS*	4	0.38	1.00
511	*EPS15*	2	0.38	1.00
512	*AMT*	2	0.38	1.00
513	*LRP3*	3	0.38	1.00
514	*CUX1*	2	0.38	1.00
515	*NCAPD2*	2	0.38	1.00
516	*KIAA1586*	2	0.38	1.00
517	*RBBP6*	2	0.38	1.00
518	*PDE10A*	2	0.38	1.00
519	*DAAM2*	3	0.39	1.00
520	*WDR66*	2	0.39	1.00
521	*PTPN14*	2	0.39	1.00
522	*ABCB8*	2	0.39	1.00
523	*AP1G2*	2	0.39	1.00
524	*SIDT1*	2	0.39	1.00
525	*TRPS1*	2	0.39	1.00
526	*RFC1*	2	0.39	1.00
1036	*WNT11*	2	1.00	1.00
1037	*UTP20*	4	1.00	1.00
1038	*MEGF6*	3	1.00	1.00
1039	*TRIM65*	2	1.00	1.00
1040	*USP45*	2	1.00	1.00
1041	*RIF1*	2	1.00	1.00
1042	*SLC12A8*	2	1.00	1.00
1043	*SLC2A2*	2	1.00	1.00
1044	*WNK4*	2	1.00	1.00
1045	*BAZ2B*	4	1.00	1.00
1046	*C4orf51*	2	1.00	1.00
1047	*CYP24A1*	3	1.00	1.00
1048	*SNTG2*	2	1.00	1.00
1049	*TMEM43*	2	1.00	1.00
1050	*SULT1C3*	2	1.00	1.00
1051	*WWTR1*	2	1.00	1.00
1052	*TIGD1*	2	1.00	1.00
1053	*BTN2A1*	2	1.00	1.00
1054	*CAPN5*	2	1.00	1.00
1055	*CCDC96*	2	1.00	1.00
1056	*FREM2*	3	1.00	1.00
1057	*ZNF621*	2	1.00	1.00
1058	*OR2T33*	2	1.00	1.00
1059	*UGT1A4*	2	1.00	1.00

527	*PLEKHA8*	2	0.39	1.00	1060	*PLEKHA6*	3	1.00	1.00
528	*FBN3*	5	0.40	1.00	1061	*CAPN13*	2	1.00	1.00
529	*ATP6V1B1*	2	0.40	1.00	1062	*CCDC146*	2	1.00	1.00
530	*PCLO*	2	0.40	1.00	1063	*CCHCR1*	2	1.00	1.00
531	*DENND2C*	2	0.40	1.00	1064	*CDHR5*	2	1.00	1.00
532	*FER1L6*	2	0.40	1.00	1065	*XDH*	2	1.00	1.00
533	*SMG6*	3	0.40	1.00	1066	*RNF207*	2	1.00	1.00

Bold font indicates statistical significance.

nVariants represents the number of variants used in the analysis.

Corrected *P* was corrected by Bonferroni correction.

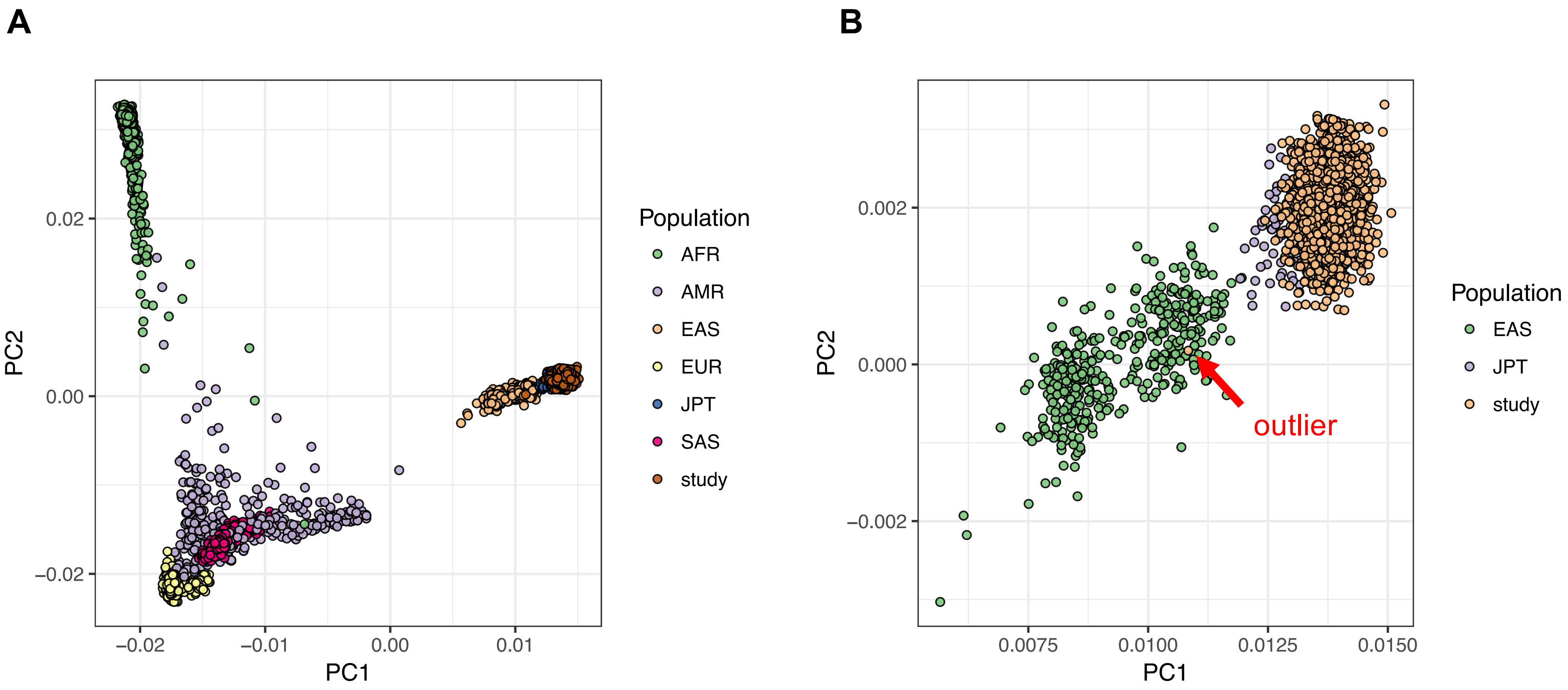

Fig. S1. Principal component analysis (PCA) plots used for ancestry inference and outlier removal. (A) PCA including multiple reference populations (AFR, African; AMR, American; EAS, East Asian; EUR, European; JPT, Japanese; SAS, South Asian). (B) PCA focusing on East Asian populations.
